# Pharmacist-assisted implementation of guideline recommendations for QTc monitoring during psychopharmacotherapy - a prospective, randomized feasibility study

**DOI:** 10.3389/fpsyt.2025.1606497

**Published:** 2025-08-28

**Authors:** Katharina Wien, Jan Philipp Klein, Niels Rodenstein, Julia Vogler, Roland Richard Tilz, Julia Thern, Stefan Borgwardt

**Affiliations:** ^1^ Hospital Pharmacy, University Hospital Schleswig-Holstein, Lübeck, Germany; ^2^ Department of Psychiatry, Psychosomatics and Psychotherapy, University of Lübeck, Lübeck, Germany; ^3^ Center of Brain, Behavior and Metabolism, University of Lübeck, Lübeck, Germany; ^4^ Department of Rhythmology, University Heart Center Schleswig-Holstein, Lübeck, Germany

**Keywords:** psychopharmacotherapy, QTc-prolongation, monitoring, guideline implementation, clinical pharmacists, interdisciplinary collaboration

## Abstract

**Introduction:**

Many psychotropic drugs potentially prolong the QTc interval. The aim of this study was to investigate the feasibility of the pharmacist-supported implementation of a new local guideline for QTc monitoring at a psychiatric department and to gather preliminary data on the effectiveness of this intervention.

**Methods:**

The study was conducted as a prospective, randomized-controlled feasibility study at the inpatient service of a department of psychiatry. In the intervention group (IG), guideline-based monitoring of QTc intervals was supported by a clinical pharmacist. The control group (CG) was solely monitored by the treating physicians. The primary effectiveness outcome was the mean change in the QTc interval between admission (T_0_) and 30 days after admission (T_1_). The implementation ratio of guideline recommendations and the acceptability of the pharmacist support based on an online employee satisfaction survey were assessed as secondary outcomes.

**Results:**

160 patients were recruited and randomly allocated to the IG (*n* = 75) or CG (*n* = 85). A total of 102 patients completed the trial (IG: *n* = 42, CG: *n* = 60). There was no significant difference in the mean change of the QTc interval from T_0_ to T_1_ between the two groups (*p* = 0.582). Guideline recommendations were significantly more often implemented in the IG than in the CG (78% vs. 37%, *p* = 0.004). 4 out of 15 (26.7%) physicians and 9 out of 45 (20%) nurses working on the wards responded to the online survey. Physicians and nurses were very satisfied with the pharmaceutical support.

**Discussion:**

Pharmacist support of the implementation of a new guideline for QTc monitoring led to a higher rate of uptake of guideline recommendations but did not have a significant effect on the QTc interval. The responding physicians and nurses were very satisfied with this intervention. Future research using an adapted study design would be required to assess guideline adherence as the primary outcome and, secondarily, determine the effect of pharmacist support on cardiac outcomes.

**Clinical trial registration:**

German Clinical Trials Register: DRKS00033127, https://drks.de/search/de/trial/DRKS00033127.

## 1 Introduction

Different drugs, including many psychopharmacologically active agents such as citalopram, haloperidol and amitriptyline, may prolong the QTc interval. The *Arizona Center for Education and Research on Therapeutics* (AZCERT) provides a list of drugs with a risk classification for QT prolongation on the web-based database *CredibleMeds*, which can be accessed free of charge (**Supplementary Table 1**) (1). At QTc intervals over 500 ms, patients are at an increased risk for developing torsades de pointes (TdP) arrhythmia which might lead to ventricular tachycardia and sudden cardiac death (2). Therefore, an increase of the QTc interval to more than 500 ms or by more than 60 ms since the start of the respective drug must be followed by an immediate change in pharmacotherapy (2). Furthermore, a QTc interval of more than 450 ms in men and 460 ms in women is considered the limit above which the patient’s ECG should be monitored more thoroughly (3–6).

Patients’ risk for QTc prolongation increases in the presence of several risk factors. Different risk assessment tools for QTc prolongation including various risk factors have previously been validated and may be used to identify patients with an increased risk for QTc prolongation (3–5). The *modified RISQ-PATH Score* has been validated in hospitalized psychiatric patients (4). It includes all prescribed QT-prolonging drugs, weighted according to their individual risk of QTc prolongation based on AZCERT categories and several risk factors relevant to psychiatric patients such as biological sex, age, smoking status, a body mass index above 30 kg/m², thyroid disturbances, diabetes, potassium level below 3.5 mmol/L, and increased creatinine level (> 1,15 mg/dL [men], > 0,95 mg/dL [women]) (4).

One or more potentially QT-prolonging drugs are frequently prescribed for psychiatric patients. In a large naturalistic study in 10 German psychiatric hospitals (OSA-PSY), one to eight QT-prolonging drugs were prescribed in 83% of 27396 patient cases (7). In 50% of cases, combinations of two or more QT-prolonging drugs were prescribed (7). Other studies identified simultaneous prescriptions of at least two QT-prolonging drugs in 7.3% (8), 51.7% (9), and 70.7% (10) of patients. In addition, prevalence rates of QTc times ≥ 500 ms or an increase in QTc time of more than 60 ms under therapy with QT-prolonging drugs between 0.9% and 2.6% were identified in various settings (10–13), including cases of serious cardiac events with a fatal outcome (10, 13). Although severe cases of drug-induced QTc prolongation occur seldomly, they are preventable using risk-adapted protocols for QTc monitoring under drug therapy.

Clinical guidelines have been developed for QTc monitoring in psychiatric patients, which are intended to support physicians in the further diagnostic and therapeutic procedure (14, 15). The goal of these guidelines is to improve drug therapy safety and at the same time ensure that patients are not denied necessary psychopharmacological therapies (14, 15). In a recent review by Peters et al. (2022) on the implementation of clinical guidelines, four interventions involving pharmacists were cited (16). However, none of them addressed pharmaceutical support for ECG monitoring during psychopharmacotherapy. In pediatric patients, the pharmaceutical recommendation of ECG monitoring using the protocol established in the clinic led to a significant increase in the rate of ECG monitoring from 47.8% to 100% (17). A US-American study demonstrated that pharmacist involvement in QTc interval monitoring utilizing a uniform protocol may improve the appropriateness of ECG utilization in patients in an acute care inpatient psychiatric hospital (18). On one of the study sites in New Jersey, appropriate ECG utilization increased by 25.5% after implementation of a standardized monitoring protocol (*p* = 0.0172) and appropriate omission of ECG utilization in patients with a low risk for QTc prolongation improved by 26% (*p* = 0.00001) (18). The participants’ change in QTc intervals was not assessed as an outcome variable (18). Several other interventional studies have demonstrated that pharmacists are able to support drug treatment and to prevent and solve drug-related problems in psychiatric inpatients, especially through medication reconciliation, medication reviews and multidisciplinary ward rounds (19).

Prior to this study, a local guideline was created at the psychiatric department of the University Hospital Schleswig-Holstein in Lübeck, Germany, to standardize ECG control intervals in psychiatric patients receiving psychopharmacotherapy with consideration of patient-specific risk factors for QTc prolongation. It was unclear whether support by a clinical pharmacist (CP) may improve the implementation of new guideline recommendations regarding ECG controls under psychopharmacotherapy.

Therefore, the aim of this study was to investigate the feasibility of guideline-based monitoring of the QTc interval under psychopharmacotherapy supported by a clinical pharmacist and to gather preliminary data on the effectiveness of this pharmaceutical intervention.

## 2 Methods

The results of this study are reported according to the CONSORT Statement (20). This report grouped patients as men or women based on their biological sex, regardless of their social gender.

### 2.1 Trial design

The study was conducted as a monocentric, prospective, interventional, randomized-controlled feasibility study with two parallel study arms for seven months between January and July 2024. One arm was the intervention group (IG), in which monitoring of the patients’ QTc intervals based on a local guideline was supported by one CP who could order ECG and laboratory checks. The second arm was the control group (CG), in which only the treating physicians conducted guideline-based monitoring of the patients’ QTc intervals (standard of care). Patients who gave informed consent to participate in the study were allocated to the IG or CG at a ratio of 1:1.

An online employee survey was designed *de novo* by the CP who supported the guideline-based QTc monitoring. The survey questions were validated by a senior psychiatrist and a senior pharmacist. The online survey was approved by the staff council of the Center for Integrative Psychiatry and conducted among all nurses and physicians working on the study wards at the psychiatric facility six months after the start of the study. The study protocol was approved by the responsible Ethics Committee of the Medical Faculty at the University of Lübeck (2023-705_1). The study was prospectively registered at the German Clinical Trials Register (DRKS00033127).

### 2.2 Participants

#### 2.2.1 Inclusion and exclusion criteria

Patients were included in the study if they were admitted to one of the three study wards or to the study day clinic at the psychiatric hospital between January and June 2024, were 18 years or older, had an expected length of hospital stay of at least 30 days, were prescribed at least one QT-prolonging drug (*known, possible* or *conditional risk* according to CredibleMeds) during the first 30 days after their hospital admission and agreed to participate in the study by signing the informed consent waiver.

Patients were excluded before the informed consent discussion with the CP if the nursing staff on the ward considered their condition after admission to be unfit for an informed consent discussion, if the patient was afraid of ECGs, or if the patient did not speak German. Patients were excluded from the study after initial enrolment if their actual length of hospital stay was shorter than 30 days, if they were not prescribed any QT-prolonging drugs, if a QT-prolonging drug was only taken as needed for less than 15 days, or if the first regularly taken QT-prolonging drug was prescribed less than seven days before T_1_. Furthermore, patients were excluded from the study if no ECG was recorded during the first seven days after admission or if they withdrew consent to participate in the trial.

As a realistic time point for ECG recordings to compare the study participants at T_1_ was needed, an observation period of 30 days was chosen because of the mean length of hospital stay of 40 days in a previous study conducted on two of the study wards (21). Taking the limited ECG resources into account, this allowed for ECG measurements in most of the study participants. Furthermore, the local guideline recommends ECG controls seven days after starting a new potentially QT-prolonging drug with a known or possible risk for QT prolongation or a conditional risk in the presence of at least one additional risk factor. Therefore, if a potentially QT-prolonging drug was only taken for less than seven days, no ECG control was required and hence, respective study participants were excluded from the analysis.

Eligible patients were approached at admission. Before ordering the ECG 30 days after admission (T_1_), the CP checked again for the presence of exclusion criteria. If present, the CP ordered no further ECG control and the patient was excluded from the final analysis.

Study participants were allowed to take other, non-psychotropic QT-prolonging drugs (e.g., antiarrhythmics, antibiotics, furosemide, hydrochlorothiazide, pantoprazole). These drugs were systematically documented with their corresponding AZCERT categories both at admission or if they were newly prescribed during the study period, and they were evaluated within the individual patient’s risk profile based on the risk factors defined in the local guideline (**Supplementary Table 2)**. Therefore, consistent with the local guideline, the prescription of non-psychotropic QT-prolonging drugs could have influenced the ECG control intervals or could have made dose adjustments necessary in case pharmacokinetic drug-drug interactions (DDIs) were present.

#### 2.2.2 Setting

This study was conducted at the Center for Integrative Psychiatry (ZIP gGmbH) in Lübeck, a subsidiary of University Hospital Schleswig-Holstein in Lübeck, Germany, and a tertiary care center for the treatment of mental health conditions. Overall, the psychiatric clinic operates six wards, two day clinics, an outpatient center, and a sleep laboratory. This study was conducted on three wards and in a day clinic with a maximum capacity of 106 beds, which mainly treat patients with affective, anxiety and obsessive-compulsive disorders and psychosomatic disorders. The wards and day clinic were chosen for the study because they used the same protocol for ECG recording and evaluation, which had been established in the previous year: ECGs were recorded on two ECG devices operated by trained nurses in the admission building and evaluated by physicians from the department of rhythmology. In accordance with DIN EN 62353, the functionality of the two ECG devices is checked once a year.

Prior to the study, regular plausibility checks by CPs, including the study CP, of electronic medication charts in the computerized physician order entry (CPOE) system of all psychiatric inpatients and day clinic patients at the psychiatric hospital had been established in routine care. Between October 2021 and May 2022, the study CP already supported the introduction of the CPOE system at the psychiatric hospital (21). Since October 2022, she took part in senior physician rounds addressing psychopharmacotherapy on two of the study wards. At the time of this study, the CP participated in the senior physician rounds on one study ward every four weeks.

In December 2023, a local guideline for QTc monitoring, developed mainly by a senior psychiatrist and a senior cardiologist with the support of the study CP, was introduced at the psychiatric clinic through the hospital’s quality management system. In addition, the study CP gave educational classes on the new guideline to the nurses and physicians working on the study wards in December 2023 and January 2024 before the start of the trial. Due to the parallel study design, the same physicians and nurses could have been involved in the treatment of patients both in the CG and in the IG. Between January and June 2024, the study CP approached eligible patients and invited them to participate in the study after an informative discussion during their first week after admission to one of the study wards.

### 2.3 Interventions

**Figure 1:**
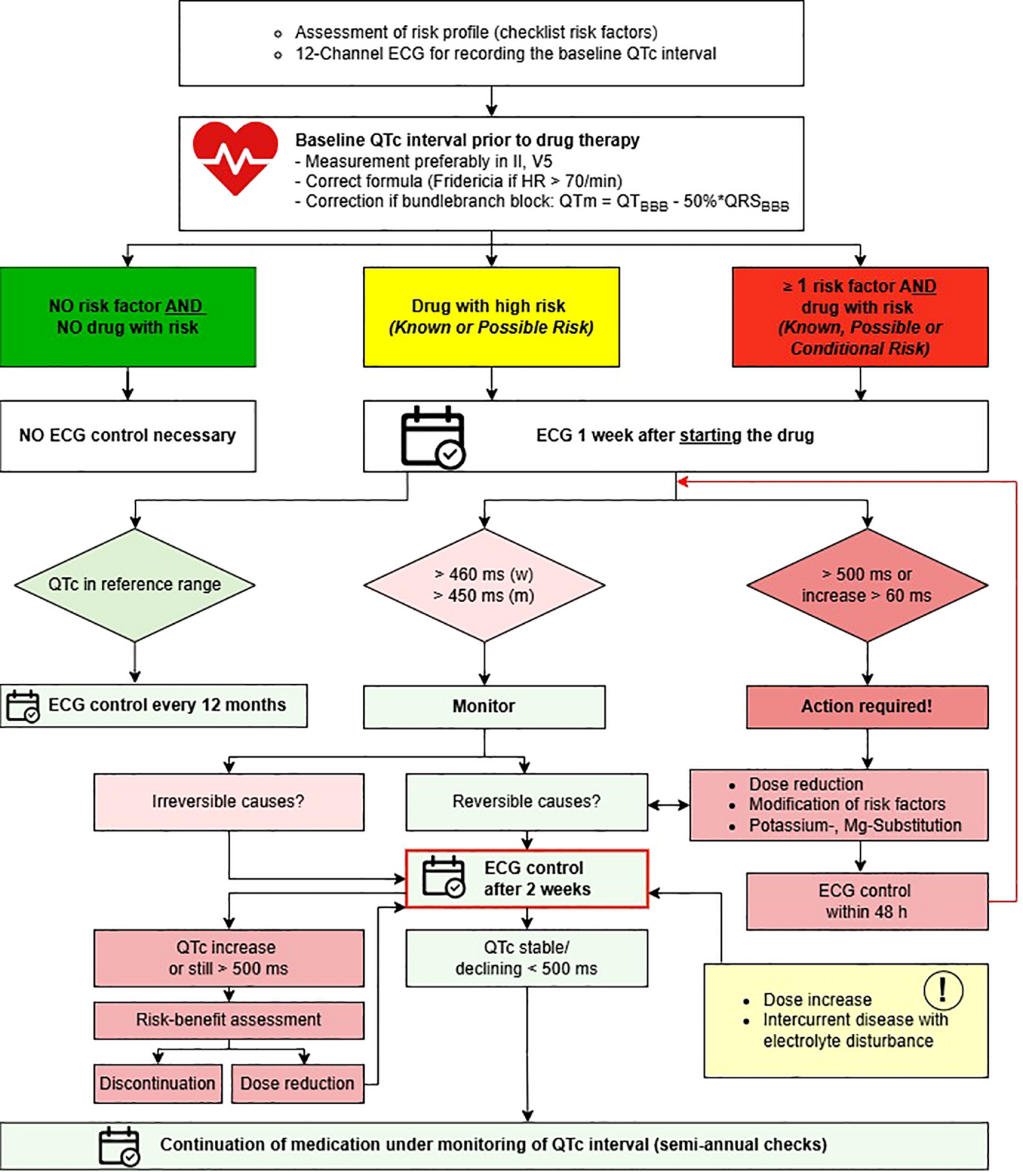
Algorithm for ECG monitoring under psychopharmacotherapy, translated from the internal local guideline at the Center for Integrative Psychiatry, Lübeck. *ECG*, electrocardiogram; *HR*, heart rate; *QTm*, modified QT interval in the presence of bundle branch block; *BBB*, bundle branch block; *Mg* magnesium. Risk categories based on AZCERT classification (1).

The algorithm for ECG monitoring under psychopharmacotherapy from the local guideline includes recommendations for ECG control intervals. Its translated version is presented in **Figure 1**. The study CP was trained in ordering ECG controls and laboratory checks by the senior psychiatrist involved in guideline creation and by the head nurse in December 2023. Prior to the start of the study, templates for ordering ECGs and laboratory controls in the electronic patient files (EPF), created by the CP based on the algorithm, were approved by the study team.

In the IG, a CP supported guideline-based monitoring of the patients’ QTc intervals. When potentially QT-prolonging psychopharmacologic drugs were newly prescribed or when their dosages were increased, the CP ordered guideline-based ECG controls in the EPF if they had not been planned accordingly by a physician. Due to technical difficulties, ECG controls were ordered via e-mail to the nurses and physicians for patients in the day clinic instead of in the EPF. Based on the calculated QTc intervals, the CP gave recommendations to the treating physicians on dose adjustments, controls of drug concentrations in blood, or supplementation of electrolytes in written form.

In the CG, the patients’ QTc intervals were monitored solely by the treating physicians. As a safety measure, the CP was allowed to intervene if a drug with a *Known* or *Possible Risk* for QT prolongation based on AZCERT (**Supplementary Table 1**) was newly prescribed to a patient with a baseline QTc interval higher than 500 ms, if the QTc interval increased to more than 500 ms or by more than 60 ms during treatment with a QT-prolonging drug and if the treating physician did not decrease the dosage or stop the potentially responsible drugs.

As established in routine care, CPs checked the plausibility of electronic medication charts in the computerized physician order entry (CPOE) system of all psychiatric inpatients at the psychiatric clinic. During the study period, pharmaceutical recommendations were written in the medication charts in cases of clinically relevant DDIs or if prescribing errors were suspected. However, notes regarding DDIs between QTc-prolonging drugs (QT-DDIs) were not written in the medication charts, so they would not interfere with the study CP’s interventions.

### 2.4 Outcomes

The study’s primary outcome was the change in the QTc interval in the observation period between T_0_ (QTc interval in ECG at admission) and T_1_ (QTc interval in ECG 30 days after admission).

As a *standard of care*, the treating physicians monitored participants in both groups for changes in their QTc intervals following changes in drug treatment, based on control intervals for 12-channel ECGs recommended in the local guideline.

Within the first seven days after admission, an ECG was recorded in all participants to assess the patients’ baseline QTc intervals (T_0_). Furthermore, the risk profile based on risk factors listed in the local guideline (**Supplementary Table 2**) and accordingly, the recommended ECG control intervals adapted to the individual patient’s risk for QT prolongation were determined by the CP at time point T_0_.

ECGs recorded in patients at the psychiatric hospital were routinely interpreted by physicians from the Department of Rhythmology at the University Hospital Schleswig-Holstein in Lübeck. As defined in the local guideline, the QT intervals recorded in ECGs were corrected using Bazett’s formula (22) for patients with heart rates (HR) below 71 beats per minute (bpm). In patients with HRs of 71 bpm or higher, QT intervals were corrected using Fridericia’s formula (23).

In both groups, if an ECG was recorded, the CP documented QTc intervals of all study participants one week after starting a new QT-prolonging drug. For all study participants, an ECG control was ordered 30 ± 8 days after admission (T_1_), and the recorded QTc intervals were documented. Furthermore, in addition to the risk profile created based on the local guideline at admission, the modified RISQ-PATH score (**Supplementary Table 3**) was calculated for all participants at time points T_0_ and T_1_ for evaluation purposes. It was not used during the intervention.

An online questionnaire including 13 short multiple-choice or single-choice questions and four voluntary free-text questions was used to assess the physicians’ and nurses’ satisfaction with the pharmaceutical support, their desire to continue pharmaceutical support measures, and the availability of personnel- and equipment-related resources for guideline-based ECG writing. The survey was designed *de novo* by the CP who supported the guideline-based QTc monitoring and the questions were validated by a senior psychiatrist and a senior pharmacist. The survey questions asked employees to rate e.g. the usefulness of the pharmacist’s recommendations for the treatment of their patients (*Very useful: 1; Useful: 2; Partly useful: 3; Mostly not useful: 4; Not useful: 5*), the satisfaction with the interdisciplinary treatment and pharmaceutical contribution (*Very satisfied: 1, Satisfied: 2, Rather not satisfied: 3, Not satisfied: 4*), the desire to continue the pharmaceutical support after the trial, and the availability of personnel and material resources for guideline-based ECG monitoring (*Sufficient: 1, Rather sufficient: 2, Rather insufficient: 3; Insufficient: 4*). The survey questions relevant for this report are presented in **Supplementary Table 8** (multiple- or single-choice questions) and **Supplementary Table 9** (free text questions). Six months after the start of the study, the study CP distributed the questionnaire electronically via e-mail invitations to all physicians (*n* = 15) and nurses (*n* = 45) working on the study wards and the day clinic. The study CP sent two e-mail reminders to all potential respondents after 14 and 25 days. All responses received within four weeks following the dispatch of the questionnaire were scrutinized.

Secondary outcomes were the number of patients who experienced QTc prolongation during the study period with QTc intervals over 450 ms (men) or over 460 ms (women), over 500 ms, or an increase by more than 60 ms, the implementation ratio of guideline recommendations, the occurrence of cardiac events, the satisfaction of psychiatric physicians with pharmaceutical support, and the physicians’ desire to continue pharmaceutical support.

### 2.5 Data collection

EPFs and medication charts from the CPOE system were screened prospectively by the study CP who also delivered the intervention. Data were collected pseudonymously in an Excel sheet and anonymized after all analyses were completed.

The CP checked the respective QTc intervals for all study patients in the findings of the Department of Rhythmology in the EPFs and documented the time required to obtain an ECG verification. The occurrence of cardiac events was documented based on follow-up entries and discharge letters from the EPFs. Cardiac events were defined as cardiac arrhythmias, including TdP tachycardia (15).

The implementation ratio of the guideline recommendations was recorded as the number of implemented recommendations divided by the number of applicable guideline recommendations for each patient individually. For this purpose, the CP documented all recommendations that could be derived from the local guideline based on the patient’s risk profile. The CP checked whether each recommendation was implemented and recorded “1” for all implemented recommendations and “0” for all unimplemented recommendations.

### 2.6 Data appraisal

A standardized Excel spreadsheet with pre-prepared drop-down lists was constructed utilizing the data structures mentioned above and was agreed upon by the study team. QTc intervals not corrected using the correction formula (Bazett’s or Fridericia’s) specified for the respective HR in the local guideline were again corrected by the study team using the correct formula. In the analysis of the change in QTc intervals, only the QTc intervals calculated with the recommended correction formulas were included. The same study CP recorded all data and then analyzed it with support from the study team.

### 2.7 Sample size

To date, no study has reported an effect size on the CP-supported guideline implementation for ECG monitoring under psychopharmacotherapy or any related interventions. Therefore, a practical approach was used to estimate a realistic study size for this feasibility study.

In a retrospective analysis of medication data retrieved from the CPOE system of patients admitted to one of the three study wards and the study day clinic between January and June 2023, 205 patients received at least one psychotropic drug with a Known or Possible Risk for QT-prolongation according to AZCERT for at least seven days. They were co-prescribed with a mean of 1.4 ± 0.7 QT-prolonging psychotropic drugs (median: 1, range: 1-4).

Since similar studies had a dropout of 20% (24), it was estimated that 80 participants (80%) could be enrolled in the IG and CG, for a total study size of 160 participants, during the recruitment phase of six months between January and June 2024.

### 2.8 Randomization

Patients were allocated to the IG or CG at a ratio of 1:1 by blocked randomization with randomly selected block sizes stratified by the four study wards. To ensure allocation concealment, the randomization lists were generated in advance and only available to a study assistant who was not involved in conducting the study. After the CP enrolled each participant, the study assistant informed the CP of the respective group allocation. The CP then assigned participants to the interventions.

Due to the parallel study design and the nature of this interventional study, blinding the treating physicians or the study pharmacist was impossible. However, the participating patients were not informed of their group allocation.

### 2.9 Statistical methods

Data were analyzed using the jamovi software (The jamovi project (2021). jamovi. (Version 2.6.13) [Computer Software]. Retrieved from https://www.jamovi.org). The significance level was set to 0.05. Patient characteristics, such as age, sex, and primary diagnosis, are presented as median values with interquartile range (IQR) or absolute and relative frequencies. The demographic comparability of the IG and CG on admission was assessed using independent sample *t* tests for continuous variables (age, baseline QTc interval) and chi-squared or Fisher’s exact tests for categorical and discrete variables (biological sex, modified RISQ-PATH score, primary diagnosis, ward, new prescription of a QT-prolonging drug).

The mean change in the QTc interval between T_0_ and T_1_ was compared between the two groups using a two-sided Student’s *t*-test.

Due to the nature of the study, participants could meet exclusion criteria after randomization if they were not prescribed any QT-prolonging drugs. Furthermore, if no ECG was written at T_0_ or T_1_, the change in QTc interval could not simply be calculated. Therefore, a strategy to handle missing data was implemented. We assumed that missing values were mostly missing at random (MAR), but it was possible that they were missing not at random (MNAR). An intention-to-treat (ITT) analysis based on all patients who had received baseline assessments at T_0_ and were prescribed at least one QT-prolonging drug was conducted. For the primary analysis, missing data for QTc intervals at T_1_ were replaced by multiple imputation by chained equations (MICE) with predictive mean-matching (25) using the mice package (26) in R-environment (27). Additionally, a sensitivity analysis was conducted based on complete cases (CC) with the assumption that data were missing completely at random (MCAR).

Both in the ITT-analysis of randomized participants and in the CC analysis, the primary outcome was analyzed using a single-factor ANCOVA model with group allocation as a factor and the modified RISQ-PATH score at T_0_ as a covariate. Group differences, 95% confidence intervals (CIs), and *p*-values were provided. Furthermore, as a *post-hoc* test, Cohen’s *d* was calculated to estimate the effect size with a 95% CI.

The percentage of implemented recommendations was determined for each patient as the patient-specific implementation ratio. The implementation ratios’ mean and standard deviation (SD) were calculated separately for the IG and CG from all patient-specific implementation ratios. Furthermore, the implementation ratio of guideline recommendations was compared between groups using the chi-squared test. The number needed to treat (NNT) was calculated from the absolute risk reduction of recommendations which were not implemented in the groups.

A subgroup analysis regarding the mean change in QTc interval and the implementation ratio of guideline recommendations was conducted using the statistical methods described above for all participants who were prescribed a new QT-prolonging drug or an increase in the dosage of such a drug during the first 30 days after admission.

The results of the employee satisfaction survey were evaluated descriptively as median with IQR.

## 3 Results

### 3.1 Participant flow and recruitment

**Figure 2:**
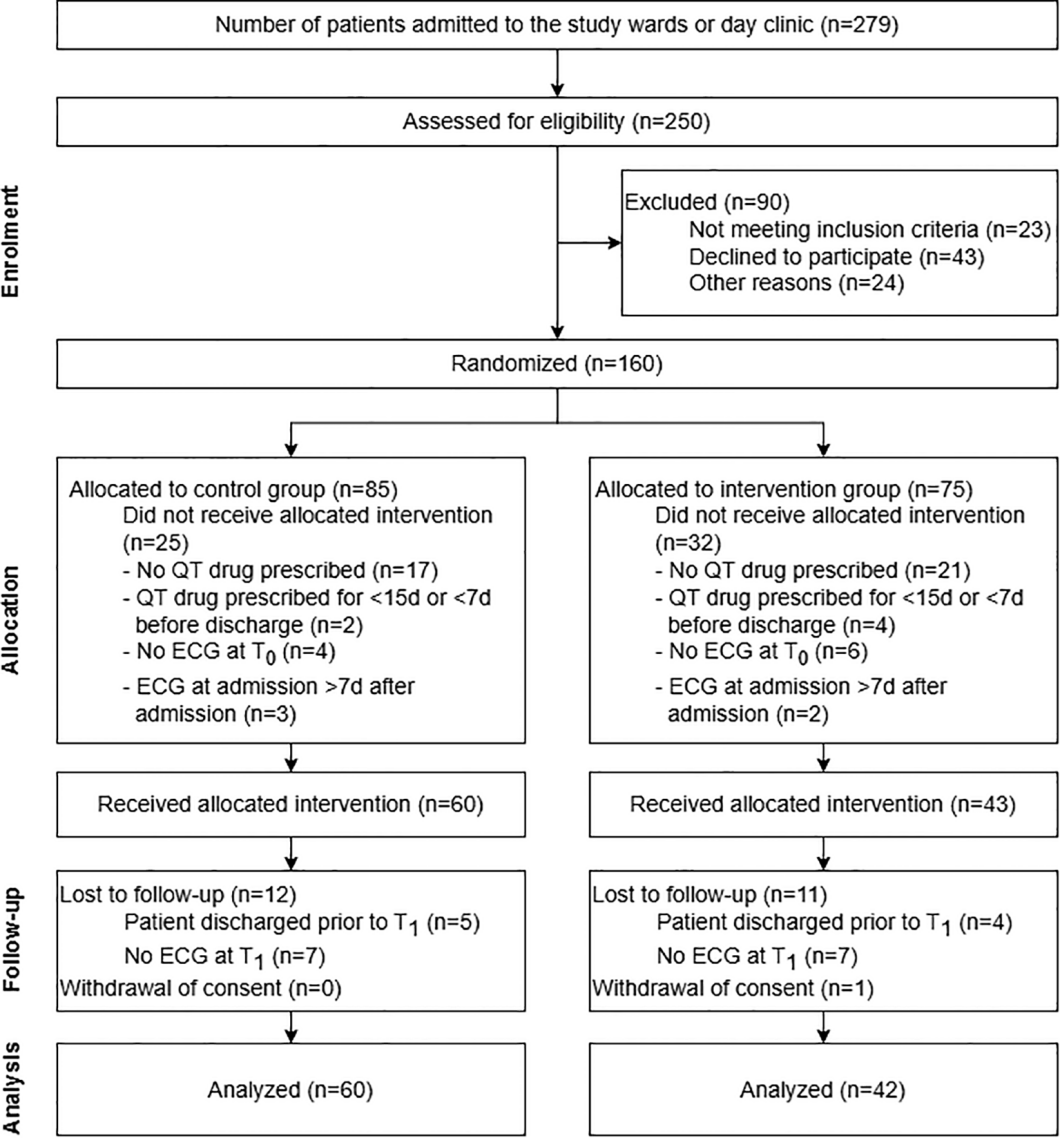
Participant flow diagram of the randomized, controlled feasibility study on pharmacist-assisted QTc monitoring from patient enrolment to data analysis. The diagram includes detailed information on the excluded participants. More than one reason why participants did not receive the allocated intervention was possible.

A participant flow diagram, including reasons for exclusion after allocation, is presented in **Figure 2**.

During the study period between January 8^th^ and June 14^th^, 2024, 279 patients were admitted to one of the study wards or the day clinic and 250 of these patients were assessed for study inclusion. 23 patients did not meet inclusion criteria at admission as they were under 18 years old (*n* = 1) and/or because their planned length of hospital stay was shorter than 30 days (*n* = 23). 24 further patients were excluded before the informed consent discussion with the CP because they were not in a fit state for an informed consent discussion (*n* = 6), afraid of ECGs (*n* = 1), did not speak German (*n* = 1), no ECG appointment was possible during the first seven days after their admission (*n* = 2), or because they were not present on the wards during the pharmacist’s visits (*n* = 14).

In total, 203 patients were invited to participate in the study. 160 patients (78.8%) agreed to participate and were randomly allocated to the CG (*n* = 85) or IG (*n* = 75). Overall, 60 patients in the CG (70.6%) and 43 in the IG (57.3%) received the allocated intervention. One patient in the IG withdrew consent to participate during the study period. 12 participants in the CG and 11 patients in the IG were either discharged prior to T_1_ or did not receive an ECG control for other reasons. For these patients, no QTc values were measured at T_1_. These missing values were computed using MICE for the ITT-analysis (**Supplementary Data Sheet 2**). A total of 48 patients in the CG (56.5% of all patients allocated to CG) and 31 patients in the IG (41.3% of all patients assigned to IG) reached the end of the study with an ECG control about 30 days after admission and were therefore included in the CC analysis. 30 and 14 participants in the CG and IG were prescribed a new or an increased dosage of a QT-prolonging drug and were consequently included in the subgroup analysis. The follow-up period terminated in July 2024 with the last ECG control visit 30 days after the last included patient’s admission.

A total of 46 patients were recruited from the first ward (CG: *n* = 24, IG: *n* = 22), 30 from the second ward (CG: *n* = 18, IG: *n* = 12), 36 from the third ward (CG: *n* = 20, IG: *n* = 16) and 47 from the day clinic (CG: *n* = 23, IG: *n* = 24). Overall, patients had a median age of 39 years, were more often women (57.9%), and were mostly admitted for the treatment of mood and affective disorders (72.3%) with major depressive disorder, recurrent severe without psychotic features (F33.2) as the most common primary diagnosis (40.9%). 76.7% of all participants (122/159) were prescribed at least one potentially QT-prolonging drug with a median of one drug in both groups. Most participants were admitted with at least one risk factor for QT prolongation based on the local guideline (mean: 1.7) and had a mean modified RISQ-PATH score of 6.2 in both groups.

There were no significant differences in demographic characteristics at admission (T_0_) and follow-up (T_1_) between the participants in the CG and IG, neither between all randomized participants, nor between the patients in the ITT-analysis, nor between those who reached T_1_. Patient demographics are presented in **Table 1** and additional information is given in **Supplementary Table 4**. Furthermore, thyroid disturbances were the only risk factor showing a difference between both groups at admission, which were more often present in the CG (17.6% vs. 5.4%, *p* = 0.026). This difference was not further evaluated in the statistical model. 4.7% of patients in the CG and 8.1% in the IG were 65 years or older (*p* = 0.516), 8.2% (CG) and 17.6% (IG, *p* = 0.095) had a known cardiac history, 17.6% (CG) and 14.9% (IG, *p* = 0.673) had bradycardia, and 71.8% (CG) and 66.2% (IG, *p* = 0.493) were already taking medication with risk for QTc prolongation. 28.2% of patients in the CG and 35.1% in the IG (*p* = 0.396) were smokers, 25.9% (CG) and 23% (IG, *p* = 0.715) had a body mass index above 30 kg/m², and 34.1% (CG) and 44.6% (IG, *p* = 0.144) had hypertension. Additional information is given in **Supplementary Table 5**.

**Table 1:** Demographic details at baseline (T_0_) and at follow-up (T_1_) of all randomized study participants (*n* = 159), of all participants who received the intervention (ITT, *n* = 102) and of those who reached T_1_ (*n* = 79).

Variable	Control_All_ (*n* = 85)	Intervention_All_ (*n* = 74)	*p*-value	Control_ITT_ (*n* = 60)	Intervention_ITT_ (*n* = 42)	*p*-value	Control_T1_ (*n* = 48)	Intervent._T1_ (*n* = 31)	*p*-value
Baseline (T_0_)									
Age [Mdn in years (IQR; R)]	39 (27; 18-75)	39.5 (25.5; 20-73)	0.431^c^	39.5 (26.3; 19-69)	48.0 (29.3; 20-73)	0.137^c^	40 (23.8; 19-67)	46 (26.5; 20-73)	0.242^c^
Gender [Female sex: *n* (%)]	50 (58.8)	42 (56.8)	0.872^b^	32 (53.3)	24 (57.1)	0.840^b^	27 (56.3)	20 (64.5)	0.492^b^
QTc interval in ms [M (± SD; R)]	407 (21.1; 366-469)	412 (24.2; 362-485)	0.162^a^	407 (21.8; 366-469)	413 (24.0; 373-485)	0.190^a^	410 (22.2; 366-469)	416 (24.6; 377-485)	0.205^a^
Main psychiatric diagnosis group			0.622^b^			0.659^b^			0.727^b^
F30–39 [*n* (%)]	59 (69.4)	56 (75.7)		42 (70.0)	31 (73.8)		37 (77.1)	23 (74.2)	
F40–48 [*n* (%)]	19 (22.4)	12 (16.2)		15 (25.0)	7 (16.7)		10 (20.8)	6 (19.4)	
*Main psychiatric diagnosis*			0.522^b^			0.896^b^			0.921^b^
Most frequent main psychiatric diagnosis: F33.2 [*n* (%)]	34 (40)	31 (41.9)		24 (40.0)	22 (52.4)		23 (47.9)	16 (51.6)	
QT-prolonging drugs at admission									
No. of QT-drugs [Mdn (IQR; R)]	1 (2, 0–5)	1 (2, 0–6)	0.311^c^	2 (1, 0–5)	2 (1, 0–6)	0.569^c^	2 (1, 0–4)	2 (1, 0–6)	0.614^c^
-w/o PRN [Mdn (IQR; R)]	1 (2, 0–5)	1 (2, 0–6)	0.341^c^	1 (1; 0-5)	2 (1; 0-6)	0.355^c^	1 (1; 0-4)	1 (1; 0-6)	0.623^c^
Risk scores									
Local guideline [M (± SD; R)]	1.7 (0.9; 0-4)	1.7 (1; 0-4)	0.152^c^	1.8 (0.8; 0-3)	2.1 (0.9; 0-4)	0.090^c^	1.8 (0.8; 0-3)	2 (0.8; 0-4)	0.182^c^
Modified RISQ-PATH score [M (± SD; R)]	6.2 (3.3; 0-17.5)	6.2 (3.4; 0-15)	0.955^c^	6.4 (3.4; 0.25-17.5)	7.0 (3.3; 0.25-14.5)	0.341^a^	6.5 (3.4; 0.25-17.5)	6.9 (3.2; 0.25-14.25)	0.360^c^
Follow-up (T_1_)									
Patients who reached T_1_ [*n* (%)]	48 (56.5)	31 (41.3)	0.081^b^	48 (80.0)	31 (73.8)	0.480^b^			
No. of QT-drugs [Mdn (IQR; R)]							2 (1.25; 0-4)	2 (1; 0-5)	0.996^c^
-w/o PRN drugs [Mdn (IQR; R)]							2 (1; 0-4)	1 (1; 0-5)	0.601^c^
Modif. RISQ-PATH score at T_1_ [M (± SD; R)]							6.6 (3.2; 0.25-14.5)	6.7 (3; 0.25-13.25)	0.706^c^
QTc interval in ms [M (± SD; R)]							412 (23.9; 350-460)	416 (22.8; 380-480)	0.402^a^

a Student’s *t*-test.

b Fisher’s exact test.

c Mann-Whitney U-test. M, Mean; Mdn, Median; IQR, Interquartile range; R, Range; QT-drug, QT-prolonging drugs; PRN, pro re nata: Drugs to be taken as needed; No., Number; w/o, without. ICD-10-WHO diagnostic classification: F30-39, Mood [affective] disorders; F40-48, Anxiety, dissociative, stress-related, somatoform and other nonpsychotic mental disorders; F33.2, Major depressive disorder, recurrent severe without psychotic features.

On admission, patients in both groups were prescribed a median of one QTc-prolonging drug (IQR: 2) with a range of 0 to 5 in the CG and 0 to 6 in the IG (*p* = 0.311). The psychotropic drug most often prescribed with a known risk for QTc prolongation was Escitalopram, which was prescribed to 11.8% of patients in the CG and 8.1% of patients in the IG at admission. Antidepressants like sertraline (conditional risk, CG: 8.2%, IG: 18.9%) and venlafaxine (possible risk, CG: 15.3%, IG: 5.4%) were prescribed as regular treatment most often. Quetiapine (conditional risk) was the drug most often prescribed overall with a potential risk for QTc prolongation in 15.3% of patients in the CG and 12.2% in the IG but only prescribed as pro re nata in some patients. Likewise, promethazine (possible risk) was prescribed in 9.4% of patients in the CG and 13.5% in the IG but mostly as pro re nata medication. Additional information on QT-prolonging psychotropic drugs prescribed at admission is presented in **Supplementary Table 6**. The somatic drug class most often prescribed was the group of proton pump inhibitors (ATC-code A02BC, CG: 15.3%, IG: 14.9%), most commonly pantoprazole (conditional risk) in 15.3% of patients in the CG and 9.5% in the IG. Additional information on QT-prolonging somatic drugs prescribed at admission is presented in **Supplementary Table 7**.

Furthermore, there were no significant differences in the percentage of patients who reached T_1_ from the different study wards (*p* = 0.056), between participants from different diagnosis groups (*p* = 0.487), based on biological sex (*p* = 0.749) or age (*p* = 0.469). However, 56.5% of all participants from the CG compared to only 41.9% from the IG reached T_1_. This difference was not statistically significant (OR: 0.56, 95% CI: 0.30, 1.04; *p* = 0.081).

### 3.2 Primary outcome: mean change in QTc interval

An overview of the mean change in the QTc intervals from the different types of analyses is presented in **Table 2**.

**Table 2:** Primary and secondary outcomes in the *ITT-analysis* (*n* = 102), the *CC-analysis* (*n* = 79), and in the *subgroup analysis* of cases with a new or increased dosage of a QT-prolonging drug (*n* = 44).

	*ITT-Analysis*			*CC-Analysis*			*Subgroup Analysis*		
Variable	CG (*n* = 60)	IG (*n* = 42)	*p*-value	CG (*n* = 48)	IG (*n* = 31)	*p*-value	CG (*n* = 30)	IG (*n* = 14)	*p*-value
Primary Outcome									
Change in QTc interval between T_0_ and T_1_ in ms [mean (± SD; range)]	3.8 (20.5; -48-63)	2.9 (18.6; -33-58)	0.821^a^	2.3 (21.8; -48-63)	0.0 (15.5; -30-33)	0.582^a^	4.7 (20.8; -34-58)	1.9 (16; -22-33)	0.665^a^
Secondary Outcomes									
Number of patients with QTc prolongation									
• QTc times > 450 ms (m) or > 460 ms (f)				3	2				
• > 500 ms				0	0				
• increase of > 60 ms				1	0				
Implementation ratio of guideline recommendations [mean (± SD; range)]				0.38 (0.43; 0-1)	0.78 (0.33; 0-1)	0.004^b^	0.38 (0.43; 0-1)	0.80 (0.35; 0-1)	0.005^b^
Occurrence of cardiac events				1	0				
Duration between ECG recording and verification by a rhythmologist in hours [median (IQR; range)]				2.8 (4.7; 5-481)	4.2 (30.2; 21-434)				

CG, Control group; IG, Intervention group; m, men; f, women; ITT, intention to treat; CC, complete case. SD, standard deviation; IQR, interquartile range.

a Student’s *t*-test.

b Chi-square test.

In the ITT-analysis, the mean QTc interval increased by 3.8 ms (SD: 20.5 ms) in the CG and by 2.9 ms (SD: 18.6 ms) in the IG between T_0_ and T_1_ (Cohen’s *d* = 0.05; 95% CI: -0.35, 0.44; *p* = 0.821; **Table 2**). In the ANCOVA model, there was no significant difference between both groups (Cohen’s *d* = 0.03; 95% CI: -0.37, 0.43; *p* = 0.886) and the modified RISQ-PATH score at T_0_ did not have a significant impact (*p* = 0.393).

In the CC analysis, the mean QTc interval increased by 2.3 ms (SD: 21.8 ms) in the CG and did not change in the IG (0.0 ms, SD: 15.5 ms) between T_0_ and T_1_ (Cohen’s *d* = 0.13; 95% CI: -0.33, 0.58; *p* = 0.582; **Table 2**). In the ANCOVA model, there was no significant difference between both groups (Cohen’s *d* = 0.10; 95% CI: -0.36, 0.57; *p* = 0.652) and the modified RISQ-PATH score at T_0_ did not have a significant impact (*p* = 0.463).

Likewise, in the subgroup analysis of patients with a new or increased dosage of a QT-prolonging drug, there was no significant difference in the mean change in QTc intervals between T_0_ and T_1_ with a mean increase of 4.7 ms (SD: 20.8 ms) in the CG and 1.9 ms (SD: 16 ms) in the IG (Cohen’s *d* = 0.14; 95% CI: -0.50, 0.78; *p* = 0.665; **Table 2**). In the ANCOVA model, there was no significant difference between both groups (Cohen’s *d* = 0.12; 95% CI: -0.54, 0.77; *p* = 0.715) and the modified RISQ-PATH score at T_0_ did not have a significant impact (*p* = 0.229).

### 3.3 Secondary outcomes

#### 3.3.1 ECG monitoring according to local guideline

A total of 175 ECGs (*CG*: 104 [59.4%], *IG*: 71 [40.6%]) were recorded in the participants during the study period. 73 of these (41.7%; *CG*: 45 [43.3%], *IG*: 28 [39.4%]) were study-related ECG controls at follow-up 30 days after admission (T_1_). A rhythmologist verified 94.3% (165/175) of the ECGs (*CG*: 96.2%, 100/104; *IG*: 91.5%, 65/71). 28% of the ECG recordings (49/175) were only verified by a rhythmologist after a notification by the pharmacist via e-mail or phone. The median duration from the time of ECG recording until verification by a rhythmologist amounted to 3.2 hours (IQR: 22.6 hours, range: 0.08–481 hours). Overall, the correct formula for the correction of the QT interval with regard to the patient’s HR according to the local guideline was used in 69% (114/165) of the verified ECGs.

Based on the local guideline, 38 and 18 recommendations for ECG controls applied to 20 and 10 study participants in the CG and IG, respectively. 36.8% (14/38) compared to 77.8% (14/18) of the recommendations were implemented in the CG and IG. The mean implementation ratio of guideline recommendations was significantly higher in the IG than in the CG (NNT = 3; *p* = 0.004; **Table 2**).

The CP intervened 31 times in the IG, 58.1% (18/31) of which were ECG orders, e.g. one week after starting a new treatment with mirtazapine in a male patient, and 22.6% (7/31) recommendations for therapeutic drug monitoring, e.g. under therapy with escitalopram before any further dose increasement (**Supplementary Figure 1**). The CP did not intervene in the CG. The pharmaceutical interventions (PIs) were implemented in 64.5% (20/31) in the IG. The types of PIs implemented by the physicians in the IG are presented in **Supplementary Figure 2**. 65% (13/20) of the implemented PIs were ECG orders.

During the study period, three and two patients in the CG and IG had QTc intervals higher than 450 ms in men or 460 ms in women and one patient in the CG had an increase in his QTc interval of more than 60 ms. None of the participants had an ECG recording with a QTc interval higher than 500 ms. There was one case of ventricular extrasystoles in the sense of ventricular bigeminy in the CG with concurrent QTc prolongation by 58 ms to 458 ms in a male patient, which was counted as a cardiac event. No cardiac event was recorded in the patients in the IG (**Table 2**).

#### 3.3.2 Employee satisfaction survey

The employee satisfaction survey was sent to all 15 physicians and 45 nurses working on the study wards and in the day clinic during the study period. 13 completed online questionnaires were received. 26.7% (4/15) of physicians and 20% (9/45) of nurses responded to the survey, contributing to a total return rate of 21.7% (13/60).

**Figure 3:**
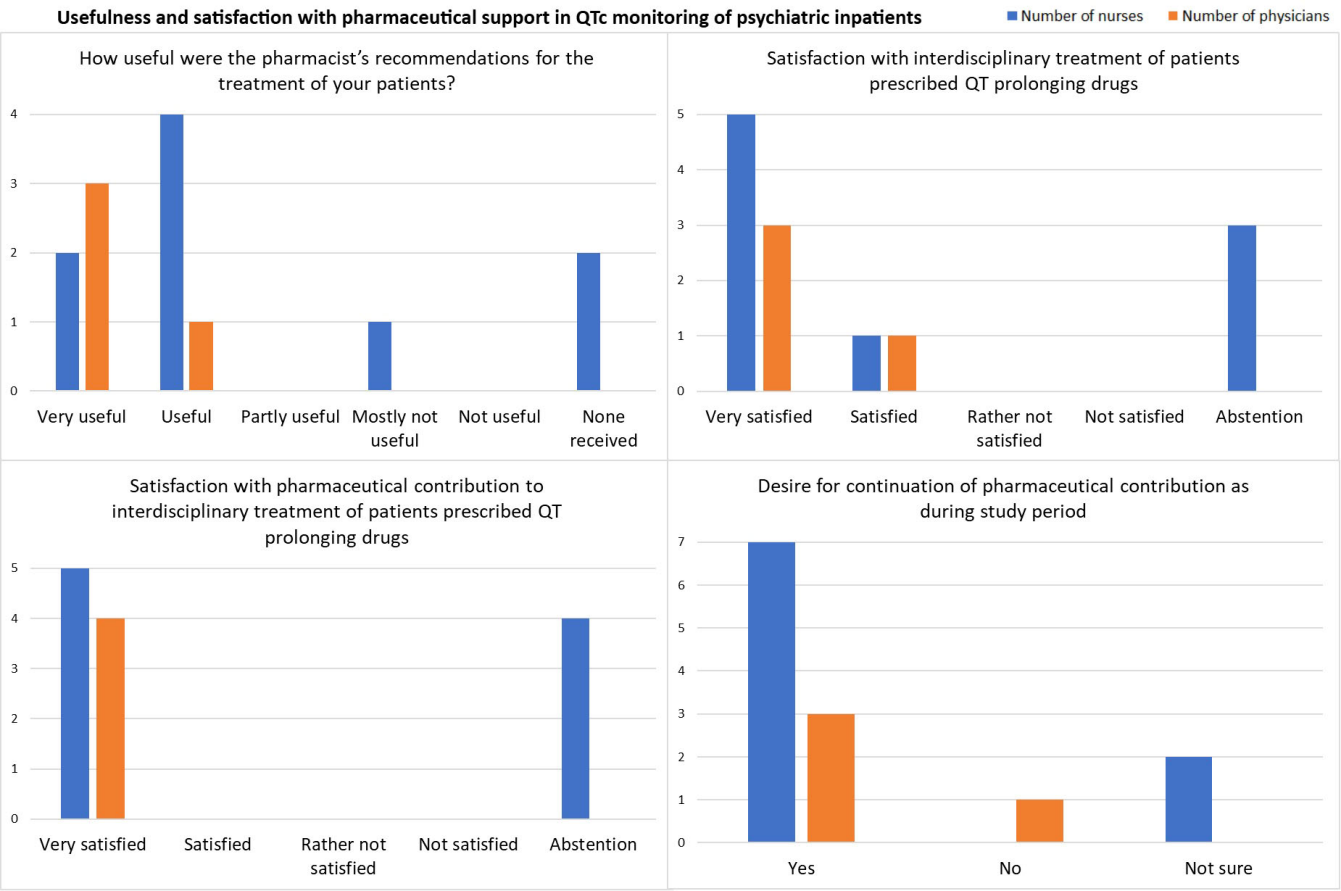
Results from the satisfaction survey in July 2024 among physicians (*n* = 4) and nurses (*n* = 9).

Overall, nurses and physicians found the pharmacist’s recommendations during the previous six months useful, and the physicians responded that they were very useful. Physicians and nurses were very satisfied with the interdisciplinary treatment of patients prescribed QT-prolonging drugs and the pharmaceutical contribution. 76.9% (10/13) of all respondents, including 75% of the responding physicians (3/4) and 77.8% of the responding nurses (7/9), expressed their desire for the continuation of the pharmaceutical contribution as tested during the intervention period (**Figure 3**, **Supplementary Table 8**).

Regarding resources for ECG monitoring at the psychiatric clinic, the responses indicated that personnel resources for standardized ECG recording were rather not sufficient, while the number of nurses trained in ECG writing and the number of existing ECG devices were estimated as rather sufficient (**Supplementary Table 8**).

In the free text questions, one participant responded that he or she would like to see irregular participation of pharmacists in senior physician rounds to improve interprofessional exchange, e.g., once per quarter, and that it would be useful to have regular discussions about medication, its effects, and its usefulness in relation to patients and their diagnoses. One physician suggested that interdisciplinary collaboration in QTc monitoring during psychopharmacotherapy would improve if feedback was given on the calculation of the QTc times including which formula was used for QT-correction (**Supplementary Table 9**).

## 4 Discussion

This was the first study to investigate the feasibility and the effect of pharmacist support on the implementation of a guideline on QTc monitoring in psychiatric inpatients receiving psychopharmacotherapy in Germany. While the mean QTc interval did not change significantly, guideline recommendations were more often implemented in the pharmacist-supported group (78% vs. 37%, *p* = 0.004) with a large effect size (NNT = 3). Furthermore, none of the patients in the IG showed an increase of their QTc intervals by more than 60 ms or any cardiac events. However, in the CG, one patient had an increased QTc interval by 63 ms to 450 ms and another patient experienced a cardiac event in the form of ventricular extrasystoles in the sense of ventricular bigeminy, accompanied by concurrent QTc prolongation by 58 ms to 458 ms. There was no change in drug therapy that might have explained the QTc increase in either of the two patients. Therefore, they were judged as non-drug-related and unavoidable by the intervention. Overall, psychiatric physicians and nurses were very satisfied with the interdisciplinary treatment of patients prescribed QT-prolonging drugs and expressed their desire for the continuation of the pharmaceutical support.

The intervention only applied to patients receiving at least one potentially QT-prolonging drug (QT drug). During the study period, 76.7% of all participants (122/159) were prescribed at least one QT drug, with a median of 1.0 in all participants and 2.0 in all participants who reached T_1_, both at admission and T_1_.

In a previous study at our psychiatric clinic, at discharge of psychiatric inpatients, 21 out of 57 (36.8%) unsolved drug-related problems caused by DDIs increased the risk for QT prolongation (QT-DDIs) (21). However, a prolonged QTc interval of over 450 ms was only detected in 13.9% of patients with QT-DDIs during hospitalization (21). In comparison, fewer patients taking at least one QT drug developed QTc intervals of over 450 ms in men or 460 ms in women or an increase by 60 ms or more during our current interventional study (*CG*: 6.3%, *IG*: 6.5%). Some patients in our current study took only one QT drug and, therefore, did not experience a QT-DDI. Consequently, the percentage of patients presenting with QTc prolongation might have been higher in patients taking multiple QT drugs as opposed to just one.

Prevalence rates between 0.9% and 2.6% for QTc intervals higher than or equal to 500 ms (10–13) and of 0.8% for an increase by more than 60 ms under drug treatment with QT drugs (10) have been identified in different settings. In a large prospective study with an observation period of five years at the University Hospital in Geneva, drug-induced QTc intervals over 500 ms were identified in 0.91% of 6790 psychiatric patients, led to sudden cardiac death in five cases (0.074% of all patients) and to TdP arrhythmia in seven cases (0.10% of all patients) (13). In our study, one of the participants in the CG (2.1% of complete cases in CG) and none in the IG showed an increase in their QTc interval by more than 60 ms during the study period. Another patient in the CG developed ventricular extrasystoles, accompanied by a concurrent QTc prolongation by 58 ms (2.1% of complete cases in CG), which was counted as a cardiac event. However, there was no change in drug therapy that might have explained the QTc increase in either of the two patients. Therefore, they were judged as not drug-induced. The cardiac event in our study was a case of ventricular extrasystoles in the sense of ventricular bigeminy without severe patient harm while only sudden cardiac death or TdP were counted as cardiac events in the study from Geneva (13). Thus, it is debatable whether the ventricular bigeminy should be counted as a cardiac event. As we had defined cardiac arrhythmias as possible cardiac events beforehand in our study protocol, we did however count this case. Furthermore, our study was much smaller and the incidence of QTc prolongation and cardiac events is very rare. Therefore, the different rates might be explained by chance.

Fortunately, none of the participants in our study developed a QTc interval above 500 ms. Statistically, one case of QTc above 500 ms could have been expected in our study sample of 122 patients receiving QT-prolonging drugs, considering the reported rates from previous studies (10–13). It would be important to investigate in a larger follow-up study whether the absence of such a case is due to chance or guideline implementation.

In our patient sample, the patients’ risk profile at admission as measured by the modified RISQ-PATH score did not have a significant effect on the mean change in QTc interval neither in the ITT-analysis (*p* = 0.393), nor in the CC analysis (*p* = 0.463), nor in the subgroup analysis of patients with a new or increased dosage of a QT-prolonging drug (*p* = 0.229). Furthermore, there were no significant differences between the results calculated in the ITT-analysis and the CC analysis. Hence, the study results were not significantly changed by the drop-outs. However, it is possible that patients with a higher risk for QTc prolongation had already been pharmacologically optimized prior to the study and therefore, did not receive any drug treatment with potentially QT-prolonging drugs.

Prior to the feasibility study, the mean change in QTc intervals was chosen as the primary outcome variable. With regard to the small potential effect size of Cohen’s *d* = 0.14 (95% CI: -0.50, 0.78) in the subgroup analysis of patients with a new or increased dosage of a QT-prolonging drug, the natural intraindividual deviation of QTc intervals must be taken into account. The mean increase between T_0_ and T_1_ of 4.7 ms (SD: 20.8 ms) in the CG and 1.9 ms (SD: 16 ms) in the IG might result from intraindividual differences between two ECG recordings as normal values for standard deviation of QT intervals in adults range between 2.1 to 2.9 ms in male and between 2.3 and 3.1 ms in female patients, depending on patient age (28). The small possible change in the QTc interval does not have a clinical significance. However, it remains important to identify the few patients on QTc-prolonging drug treatments who actually experience a clinically significant increase in their QTc intervals by more than 30 to 60 ms or to a QTc interval above 500 ms. While the mean change in the QTc interval among all patients does not appear clinically important, a standardized monitoring protocol might help to identify the few patients with significantly increased QTc intervals.

While we could not show a significant effect of the pharmaceutical intervention on the change of the QTc interval, guideline recommendations were significantly more often implemented in the pharmacist-supported group (78% vs. 37%, *p* = 0.004) with a large effect size (NNT = 3). Thus, if 3 guideline recommendations on QTc monitoring apply to psychiatric patients, one more will be implemented in the pharmacist-supported group compared to standard of care where monitoring is solely conducted by physicians. In comparison, after implementation of a standardized monitoring protocol supported by pharmacist interventions in two psychiatric hospitals in New Jersey, USA, appropriate ECG utilization increased significantly by 25.5% to 77% and appropriate ECG omission improved by 26% to 95% in one of the hospitals (18). In the other clinic, no significant change in ECG utilization was found but, notably, the intervention group was much smaller (18). While it is not possible to compare the implementation of guideline recommendations in our study directly with the changes in appropriate ECG utilization in New Jersey, both studies provide evidence that pharmacists may support the implementation of protocols for standardized ECG monitoring in psychiatric patients. Furthermore, a pharmacist intervention on ECG monitoring of pediatric patients receiving multiple QTc prolonging drugs led to an improved rate of ECG monitoring from 48% to 100% (*p* < 0.001) (17). Compared to the pediatric population, the implementation rate of the recommended ECG controls was lower both in our CG and in our IG. This might be explained by organizational issues because the patients at our psychiatric department must maintain their own appointments and change buildings for ECG controls. Furthermore, a limited number of ECG appointments were available at our hospital. While these difficulties were not discussed in the report of the pediatric study (17), time constraints and occasional patient refusal were mentioned as limitations in the adult psychiatric population (18). Another aspect worth reflecting upon is the possibility that physicians assessed patients’ risks for QTc prolongation at admission and decided after the baseline ECG that further ECG monitoring would not be necessary in some patients due to their low risk. However, the effect of starting a new drug with a potential risk for QTc prolongation or increasing its dosage on an individual patient’s QTc interval is uncertain and cannot be categorically ruled out without further ECG controls. Therefore, clinical guidelines for QTc monitoring should be followed if possible. However, it might not be important to control the QTc interval exactly one week or two weeks after changing the drug therapy as suggested in our local guideline. Both clinical pragmatism and individual decisions by the treating physicians may appropriately change intervals of ECG measurements in individual patients and clinical guidelines remain suggestions to encourage best practice.

Nonetheless, guideline adherence improved significantly as a result of pharmaceutical support. Following patient safety guidelines and documentation of QTc measurements is important to ensure a safe drug treatment for psychiatric patients and to provide a rationale for future changes in drug therapy for each individual patient. Although severe cases of QTc prolongation occur seldomly, following guidelines for QTc monitoring provides treating physicians with legal insurance in cases of patient harm.

Overall, psychiatric physicians and nurses appreciated the pharmaceutical support as assessed through an anonymous online employee satisfaction survey. 21.7% of 60 employees on the study wards and the day clinic responded to the survey with relatively more physicians (26.7% of all physicians on the study wards) than nurses (20% of all nurses on the study wards). The return rate was similar to that of a previous employee satisfaction survey at our psychiatric clinic after the implementation of a CPOE system, with response rates of 31% of physicians and 19.8% of nurses (21).

A systematic review on predictors of response rates of safety culture questionnaires in healthcare calculated a mean response rate of 66.5% ± 21.0% with a range of 4.2% to 100% (29). Reasons for low response listed in the review and potentially applicable to our survey were comprehension issues with survey questions, lack of incentives (e.g. monetary payment), questionnaire length, survey fatigue, time constraints, and timing of questionnaire distribution (29). Our survey was conducted over four weeks in July during a typical vacation period which might have limited the number of responses. It included 13 short multiple-choice or single-choice questions and four voluntary free-text questions, and we expected that it would take responders about 5–10 minutes to answer the survey. Consequently, nurses and physicians should have had enough time during the four weeks to answer the survey if they wanted to.

An additional explanation for the low response rate might be that nurses or physicians did not notice the pharmaceutical interventions during the study periods. Therefore, employees might have believed that the survey was not aimed at them and thus did not respond to it. Additionally, some nurses did not respond to all survey questions. This could also be explained by the possibility that the respective nurses were not directly involved in making the ECG appointments or did not notice if the orders for ECG controls were made by the CP or a treating physician.

Nonetheless, the survey results generally point toward a good acceptance of the pharmaceutical support at the psychiatric clinic and express the employees’ desire for the continuation of interdisciplinary QTc monitoring under psychopharmacotherapy.

### 4.1 Strengths and limitations

Our feasibility study was conducted at a psychiatric clinic in a real-world setting. Therefore, the patient sample is representative of psychiatric inpatients on the study wards and in the included day clinic at our hospital. However, large sample sizes and longer observation periods are needed to study outcomes with low prevalence rates, such as QTc prolongation and related cardiac events, which carry a risk for severe patient harm but seldomly occur.

The sample size of 159 patients overall and of only 79 patients reaching the follow-up assessment at T_1_ was too small and the observation period of 30 days per patient was too short to find significant differences in cardiac outcome parameters between the groups. Our findings are therefore exploratory. The local guideline on QTc monitoring was just implemented about one month before the start of the study and the CP trained physicians and nurses on the study wards just before enrolment began. Therefore, two interventions were tested: the implementation of the local guideline in both groups and additionally, the pharmaceutical support in the IG. Furthermore, the parallel study design may have led to a contamination bias as physicians and nurses on the study wards learned more about QTc management from the CP recommendations for IG patients and then applied their knowledge to the patients on the same wards who were allocated to the CG. Consequently, this pilot study lacked sufficient power to measure a significant effect on the mean change in the QTc interval, which was chosen as the primary outcome beforehand. However, guideline adherence improved significantly in our study and the ward staff was satisfied with the intervention which showed positive results regarding feasibility.

As a further limitation, the number of patients who reached the end of the study with an ECG control 30 days after admission was limited by the resources available for ECG recording at our psychiatric hospital. If a patient missed an ECG appointment, finding a new slot in the following days was not always possible. As a result, 15 patients (CG: *n* = 7, 8.2%; IG: *n* = 8, 10.7%) had to be excluded from the analysis due to missing ECGs at T_0_. Accordingly, nurses and physicians indicated in the survey that personnel resources for standardized ECG recording needed to be increased.

Furthermore, the intervention was conducted by the same CP who was also responsible for data collection and evaluation of the study results. The suitability and implementation of guideline recommendations was not validated by a physician or nurse. This procedure may have introduced a source of performance and reporting bias. Therefore, for future studies, we recommend the validation of data collected by a CP by a senior psychiatrist or rhythmologist. Ideally, a second researcher independent of the study hospitals and staff should collect data to avoid bias.

Although there is no consensus on an ideal or average response rate (30), our employee satisfaction survey with only 13 completed questionnaires and a response rate of 21.7% of 60 invited employees appears to limit the validity of our results since a low participation rate introduces a nonresponder selection bias (30, 31). As the study CP was participating in regular senior physician rounds on one study ward, the physicians working on that ward (*n* = 5) might have been more interested in responding to the survey and might have rated the pharmaceutical support more positively, introducing a source of social desirability bias (32). A systematic review found an average response rate of 53.3% ± 24.5% over 1746 healthcare professionals’ surveys in surgery (30). The response rates differed between the survey modes, with 50.5% ± 23.3% in email studies and 45.8% ± 25.0% in web-based surveys (30). Our survey was a web-based survey with personal invitations sent out via email. Therefore, the response rate in our survey was lower than the average of surveys conducted among healthcare professionals in surgery but within the range of the corresponding response rates. Furthermore, the survey was created *de novo* for this study. For future trials, it would be preferable to use validated questionnaires if available.

Due to the nature of this feasibility study, some adjustments should be made to the study protocol before conducting a subsequent study (33). Instead of a parallel design, a consecutive design with a usual care control group without extra guideline training by a CP, followed by a wash-out period, and then by an intervention period supported by a CP would better isolate the pharmacist’s impact. An alternative design could include randomization by hospital site to avoid control and intervention patients being on the same ward and to reduce staff contamination issues. In our feasibility study, participants were allocated in a ratio of 1:1 by blocked randomization with randomly selected block sizes stratified by the four study wards to study the influence of admission to the different study wards or day clinic regarding the effectiveness of the intervention. However, more patients were allocated to the CG (*n* = 85) than to the IG (*n* = 75). The study wards did not significantly impact the mean change in QTc intervals nor the implementation ratio of guideline recommendations. To increase the chance for a group allocation in a ratio of 1:1, we recommend omitting the stratification by ward during block randomization in future randomized controlled trials (RCTs). Furthermore, we suggest assessing guideline adherence and employee satisfaction as primary outcomes and the clinical relevance of pharmaceutical support in QTc monitoring regarding cardiac outcome parameters as a secondary outcome. Based on the results of this study, only 27 participants would be required per group to attain 80% power to detect an implementation ratio of 37% vs. 78% with a two-tailed significance level of 0.05 in two independent groups. However, given the small potential effect size regarding the mean change in the QTc intervals, 1410 participants per group would be needed to attain 80% power with a significance level of 0.05 to test whether there is a difference in the mean change of the QTc intervals between two independent groups. Even if a statistically significant difference was found, it remains uncertain whether an increase of the QTc interval by 2.3 ms compared to no increase (CC-analysis) has any clinical significance.

Regarding the design of the intervention, some further adjustments should be made before another RCT. We faced some technical difficulties with the user rights to place generic orders in the EPFs. While the study CP received the right to place generic orders and use corresponding templates in the EPF, it would be challenging to equip more CPs with these rights. Furthermore, the orders by the CP were only visible on the three wards but not in the day clinic. Therefore, the CP had to order ECGs via email for participants admitted to the day clinic. Still, there was no significant impact of admission to the wards or the day clinic regarding outcome parameters assessed in this feasibility study. For future RCTs or implementation of the tested intervention in routine care, we recommend using standardized notes in the CPOE system with clear recommendations for ECG controls after a QT-prolonging drug is newly prescribed or its dosage increased. This way, all CPs would be equipped with the technical rights to assist in QTc monitoring, while the last responsibility of ordering ECGs would remain with the treating physicians.

Furthermore, in our survey, one physician asked that the correction formula used to calculate the QTc interval should always be stated in the ECG verifications by the rhythmologists. We strongly support this wish as a criterion to improve the quality of the ECG verification process at the psychiatric clinic, considering that only 69% of the verified ECG records during the study period used or named the correction formula for the patient-specific HR specified in the local guideline. This methodological deviation further limits our study results regarding the mean change in the QTc interval. The use of different correction formulas and, additionally, correcting the QT interval in patients with a right bundle branch block changes the patients’ QTc intervals. However, the CP calculated the QTc interval using the correction formula specified in the local guideline for every ECG recording with an apparently wrong use of correction formulas to ensure the validity of the QTc intervals included in the final analysis.

Before starting another study, a standard for the documentation of the ECG verifications by the rhythmologists must be implemented, including listing the QT interval measured by the rhythmologists, the patient’s heart rate, the QT correction formula used, if applicable if and how the QT interval was corrected in case of right bundle branch block, and the calculated QTc interval.

Despite the limitations, the results of this prospective study showed the feasibility of pharmacist-supported QTc monitoring under psychopharmacotherapy. Recruitment of study participants by a CP was feasible and patients were willing to participate. This paves the way for another, preferably multicentric RCT that could be conducted to study guideline adherence and employee satisfaction as primary outcomes and the clinical relevance of interdisciplinary collaboration as a secondary outcome in a larger sample of psychiatric patients over a longer period for each participant. The potential for such collaboration is promising for the future of psychiatric patient care (34, 35).

### 4.2 Conclusion

Pharmacist support of the implementation of a new local guideline for QTc monitoring led to a higher rate of uptake of guideline recommendations but did not have a significant effect on the QTc interval. The responding physicians and nurses were very satisfied with this intervention. Future research should assess guideline adherence as the primary outcome and, secondarily, determine the effect of pharmacist support on cardiac outcomes.

## Data Availability

The raw data supporting the conclusions of this article will be made available by the authors, without undue reservation.

## Supplementary material

Supplementary Data Sheet 1 Supplementary Figures and Tables, including reporting checklist for randomised trial based on CONSORT guidelines.

Supplementary Data Sheet 2 QTc values of participants at baseline and T1, including imputed values for missing data (MICE-model).
