# Supplementary material for: Pharmacist-assisted implementation of guideline recommendations for QTc monitoring during psychopharmacotherapy - a prospective, randomized feasibility study
Source: Front Psychiatry. 2025 Aug 28;16:1606497. doi: 10.3389/fpsyt.2025.1606497 (PMC12423074; doi:10.3389/fpsyt.2025.1606497)
Supplement: Supplementary Data Sheet 1 — Supplementary Figures and Tables, including reporting checklist for randomised trial based on CONSORT guidelines. [file DataSheet1.docx]

Pharmacist-assisted implementation of guideline recommendations for QTc monitoring during psychopharmacotherapy - a prospective, randomized feasibility study

Supplementary Material

# Supplementary Figures and Tables

## Supplementary Figures

**
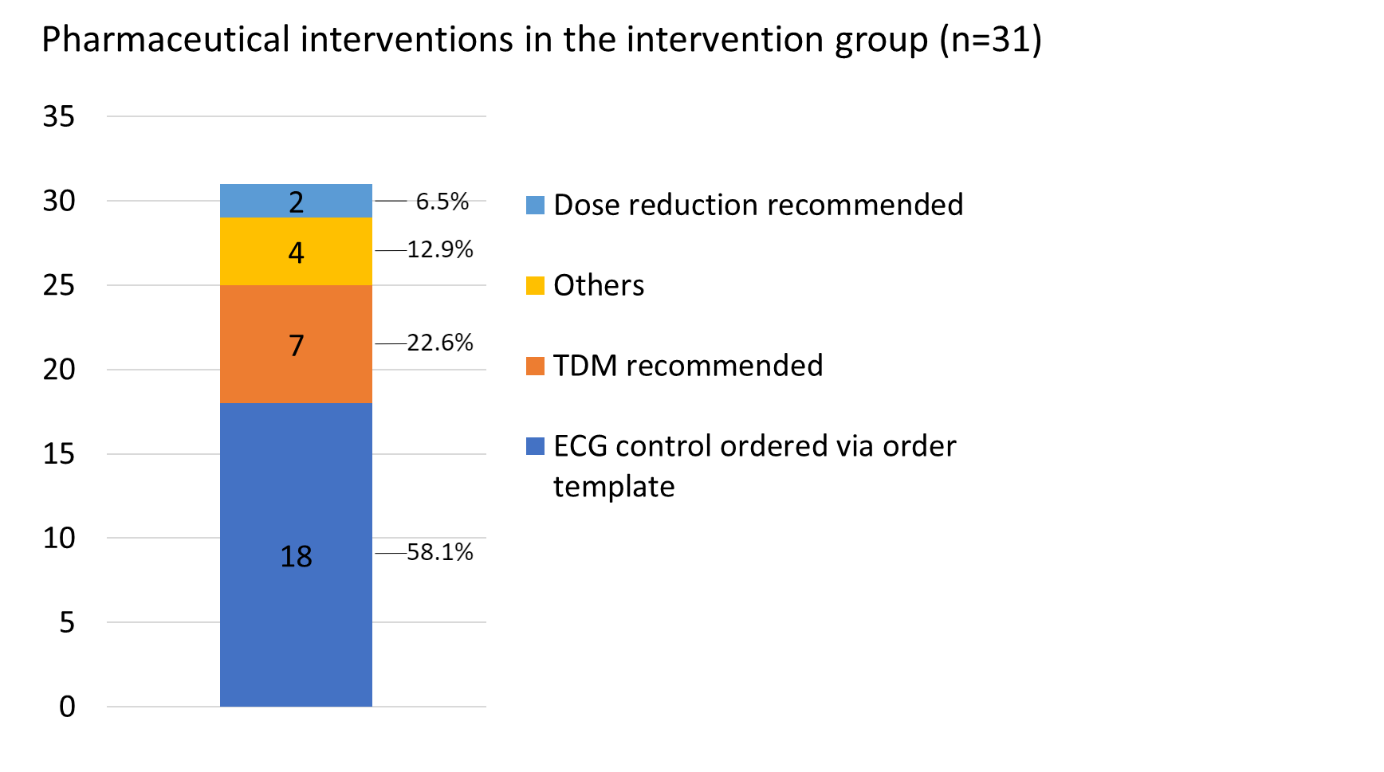
**

**Supplementary Figure 1.** Pharmaceutical interventions recommended in the intervention group.

**
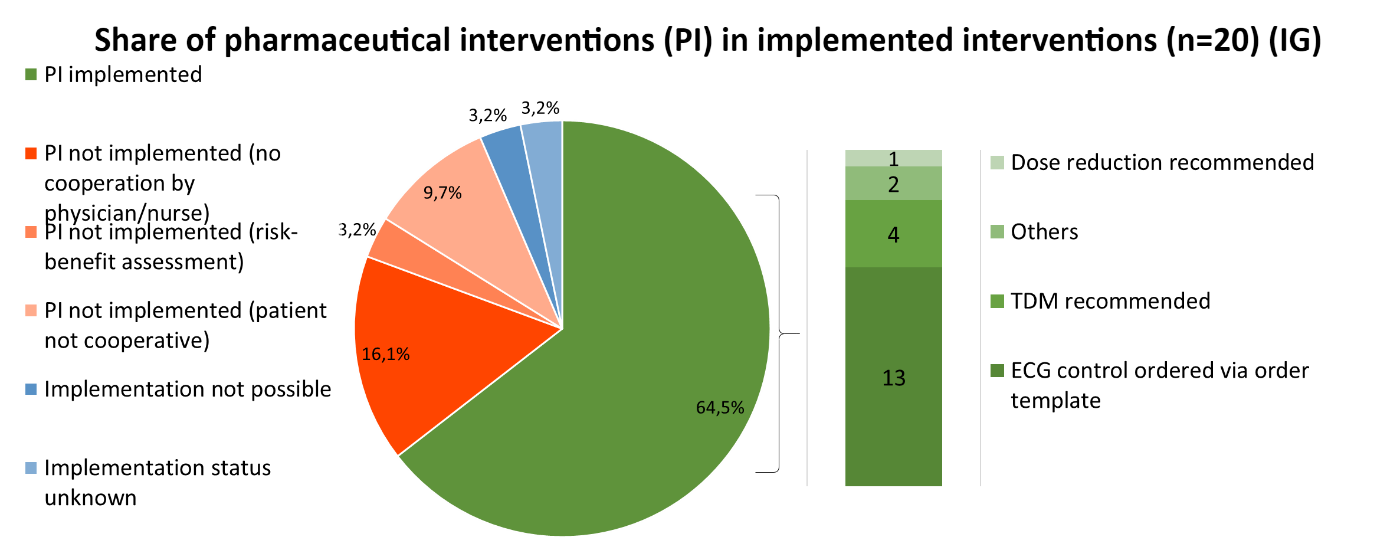
**

**Supplementary Figure 2.** Share of pharmaceutical interventions (PI) in implemented interventions in the intervention group (IG). *TDM* Therapeutic Drug Monitoring.

## Supplementary Tables

**Supplementary Table 1.** Psychotropic drugs with the potential for QT-prolongation sorted by AZCERT-categories (Woosley RL, Romero KA. QTdrugs List. (2015) www.crediblemeds.org [Accessed July 7, 2023]).

| **Known risk** | **Possible risk** | **Conditional risk** |
| --- | --- | --- |
| Citalopram | Desipramine | Amitriptyline |
| Escitalopram | Imipramine | Clomipramine |
| Chlorpromazine | Mirtazapine | Doxepin |
| Chlorprothixene | Nortriptyline | Fluoxetine |
| Droperidole | Trimipramine | Fluvoxamine |
| Haloperidol | Venlafaxine | Paroxetine |
| Levomepromazine | Aripiprazole | Sertraline |
| Sertindole | Clozapine | Trazodone |
| Sulpiride | Flupentixol | Amisulpride |
| Donepezil | Melperone | Olanzapine |
| Methadone | Pipamperone | Quetiapine |
|  | Promethazine | Risperidone |
|  | Prothipendyl | Ziprasidone |
|  | Tiapride |  |
|  | Zuclopenthixol |  |
|  | Atomoxetine |  |
|  | Levomethadone |  |
|  | Lithium |  |
|  | Zotepine |  |

**Supplementary Table 2.** Risk factors for QT prolongation listed in the local guideline. m: men. w: women.

| irreversible | - Age ≥ 65 years - Female sex - Congenital Long QT Syndrome (cLQTS) - Family history of sudden cardiac death (first degree family member) - Known cardiac history: pre-existing heart failure, myocardial hypertrophy, cardiac arrhythmias - Pre-existing QTc prolongation (> 450 ms (m), > 460 ms (w); independent of genesis) |
| --- | --- |
| reversible | - Bradycardia (heart rate at rest < 60 beats per minute) - Electrolyte disturbance (hypokalemia, hypomagnesemia, hypocalcemia) - Concomitant medication with risk for QTc-prolongation (*Supplementary Table 1*) |

**Supplementary Table 3.** Risk factors and corresponding score points contributing to the *modified RISQ-PATH* score (Vandael E, Vandenberk B, Willems R, Reyntens J, Vandenberghe J, Foulon V. Risk Management of Hospitalized Psychiatric Patients Taking Multiple QTc-Prolonging Drugs. *J Clin Psychopharmacol* (2017) 37:540–545. doi: 10.1097/JCP.0000000000000758).

| **Risk Factors** | **Points** |
| --- | --- |
| Age ≥65 years | 3 |
| Female sex | 3 |
| Smoking | 3 |
| Body mass index ≥ 30 kg/m² | 1 |
| (Ischemic) cardiomyopathy | 3 |
| Hypertension | 3 |
| Arrhythmia | 3 |
| Prolonged QTc (≥450 [men]/470 [women]ms) on a baseline ECG | 6 |
| Thyroid disturbances | 3 |
| Diabetes | 0,5 |
| Potassium ≤ 3.5 mmol/L | 6 |
| Increased creatinine (> 1,15 mg/dL [men], > 0,95 mg/dL [women]) | 0,5 |
| For each *Known risk* QT-drug CredibleMeds | 3 per drug |
| For each *Possible risk* QT-drug CredibleMeds | 0,5 per drug |
| For each *Conditional risk* QT-drug CredibleMeds | 0,25 per drug |
| **Total** | **Maximum 35 points + sum QT drugs** |

**Supplementary Table 4.** Additional demographic details of all randomized study participants (*n*= 159), of all participants who received the intervention (ITT, *n* = 102) and of those who reached T_1_ (*n* = 79).

| **Variable** | **Control_All_ (*n*= 85)** | **Intervention_All_ (*n*=** **74)** | ***p*-value** | **Control_ITT_ (*n*= 60)** | **Intervention_ITT_ (*n*= 42)** | ***p*-value** | **Control_T1_ (*n*= 48)** | **Intervention_T1_ (*n*= 31)** | ***p*-value** |
| --- | --- | --- | --- | --- | --- | --- | --- | --- | --- |
| **Baseline (T_0_)** | | | | | | | | | |
| *Wards* | | | 0.749^b^ |  | | 0.914^b^ |  | | 0.877^b^ |
| Ward 1 [*n* (%)] | 24 (28.2) | 22 (29.7) |  | 21 (35.0) | 15 (35.7) |  | 19 (39.6) | 12 (38.7) |  |
| Ward 2 [*n* (%)] | 17 (20) | 10 (13.5) |  | 10 (16.7) | 6 (14.3) |  | 7 (14.6) | 4 (12.9) |  |
| Ward 2, day clinic [*n* (%)] | 1 (1.2) | 2 (2.7) |  | 0 (0) | 1 (2.4) |  | 0 | 1 (3.2) |  |
| Ward 3 [*n* (%)] | 20 (23.5) | 16 (21.6) |  | 15 (25.0) | 10 (23.8) |  | 11 (22.9) | 6 (19.4) |  |
| Day clinic [*n* (%)] | 23 (27.1) | 24 (32.4) |  | 14 (23.3) | 10 (23.8) |  | 11 (22.9) | 8 (25.8) |  |
| *Main psychiatric diagnosis group* | | | 0.622^b^ |  | | 0.659^b^ |  | | 0.727^b^ |
| Mental disorders due to known physiological conditions (F01-09) [*n* (%)] | 2 (2.4) | 0 |  | 1 (1.7) | 0 (0) |  | 0 | 0 |  |
| Schizophrenia, schizotypal, delusional, and other non-mood psychotic disorders (F20-29) [*n* (%)] | 1 (1.2) | 1 (1.4) |  | 0 (0) | 1 (2.4) |  | 0 | 0 |  |
| Mood [affective] disorders (F30-39) [*n* (%)] | 59 (69.4) | 56 (75.7) |  | 42 (70.0) | 31 (73.8) |  | 37 (77.1) | 23 (74.2) |  |

**Supplementary Table 4 (continued).** Additional demographic details of all randomized study participants (*n*= 159), of all participants who received the intervention (ITT, *n* = 102) and of those who reached T_1_ (*n* = 79).

| **Variable** | **Control_All_ (*n*= 85)** | **Intervention_All_ (*n*=** **74)** | ***p*-value** | **Control_ITT_ (*n*= 60)** | **Intervention_ITT_ (*n*= 42)** | ***p*-value** | **Control_T1_ (*n*= 48)** | **Intervention_T1_ (*n*= 31)** | ***p*-value** |
| --- | --- | --- | --- | --- | --- | --- | --- | --- | --- |
| **Baseline (T_0_)** | | | | | | | | | |
| Anxiety, dissociative, stress-related, somatoform and other nonpsychotic mental disorders (F40-48) [*n* (%)] | 19 (22.4) | 12 (16.2) |  | 15 (25.0) | 7 (16.7) |  | 10 (20.8) | 6 (19.4) |  |
| Behavioral syndromes associated with physiological disturbances and physical factors (F50-59) [*n* (%)] | 3 (3.5) | 2 (2.7) |  | 1 (1.7) | 1 (2.4) |  | 1 (2.1) | 1 (3.2) |  |
| Disorders of adult personality and behavior  (F60-69) [*n* (%)] | 0 | 2 (2.7) |  | 0 (0) | 1 (2.4) |  | 0 | 0 |  |
| Behavioral and emotional disorders with onset usually occurring in childhood and adolescence (F90-98) [*n* (%)] | 1 (1.2) | 1 (1.4) |  | 1 (1.7) | 1 (2.4) |  | 0 | 1 (3.2) |  |

^a^Student’s *t*-test. ^b^Fisher’s exact test. ^c^Mann-Whitney U-test.

**Supplementary Table 5.** Risk factors for QTc prolongation present in patients in the control (CG; *n*= 85) and intervention groups (IG; *n* = 74) at admission.

| **Risk factors** | **Reference** | **Control_All_ (*n*= 85)** | **Intervention_All_ (*n*=** **74)** | ***p*-value** |
| --- | --- | --- | --- | --- |
| Age ≥ 65 years (%) | Local guideline and *RISQ-PATH* score | 4 (4.7%) | 6 (8.1%) | 0.516 |
| Female sex (%) | Local guideline and *RISQ-PATH* score | 50 (58.8%) | 42 (56.8%) | 0.872 |
| Congenital Long QT Syndrome (cLQTS) (%) | Local guideline | 0 | 1 (1.4%) | 0.470 |
| Family history of sudden cardiac death (first degree family member) (%) | Local guideline | 0 | 1 (1.4%) | 1.0 |
| Known cardiac history: pre-existing heart failure, myocardial hypertrophy, cardiac arrhythmias (%) | Local guideline | 7 (8.2%) | 13 (17.6%) | 0.095 |
| Pre-existing QTc prolongation (> 450 ms (m), > 460 ms (w); independent of genesis) (%) | Local guideline | 1 (1.2%) | 2 (2.7%) | 0.598 |
| Bradycardia (heart rate at rest < 60 beats per minute) (%) | Local guideline | 15 (17.6%) | 11 (14.9%) | 0.673 |
| Electrolyte disturbance   - Hypokalemia (≤ 3.5 mmol/L) (%) - Hypomagnesemia (≤ 0.7 mmol/L) (%) - Hypocalcemia (≤ 2.2 mmol/L) (%) | Local guideline  + RISQ PATH  Local guideline  Local guideline | 4 (4.7%)  0  0 | 1 (1.4%)  0  1 (1.4%) | 0.372  0.462 |
| Concomitant medication with risk for QTc-prolongation (%) | Local guideline | 61 (71.8%) | 49 (66.2%) | 0.493 |
| Smoking (%) | *RISQ-PATH* score | 24 (28.2%) | 26 (35.1%) | 0.396 |
| Body mass index ≥ 30 kg/m² (%) | *RISQ-PATH* score | 22 (25.9%) | 17 (23.0%) | 0.715 |
| (Ischemic) cardiomyopathy (%) | *RISQ-PATH* score | 2 (2.4%) | 5 (6.8%) | 0.252 |
| Hypertension (%) | *RISQ-PATH* score | 29 (34.1%) | 33 (44.6%) | 0.144 |
| Arrhythmia (%) | *RISQ-PATH* score | 2 (2.4%) | 3 (4.1%) | 0.668 |
| Prolonged QTc (≥450 [men]/470 [women]ms) on a baseline ECG (%) | *RISQ-PATH* score | 0 | 1 (1.4%) | 0.465 |
| Thyroid disturbances (%) | *RISQ-PATH* score | 15 (17.6%) | 4 (5.4%) | 0.026 |
| Diabetes (%) | *RISQ-PATH* score | 5 (5.9%) | 9 (12.2%) | 0.261 |
| Increased creatinine (> 1,15 mg/dL [men], > 0,95 mg/dL [women]) (%) | *RISQ-PATH* score | 13 (15.3%) | 11 (14.9%) | 1.0 |

^b^Fisher’s exact test

**Supplementary Table 6.** QTc prolonging psychotropic drugs and their AZCERT-categories prescribed to patients in the control (CG; *n*= 85) and intervention groups (IG; *n* = 74) at admission.

| **Variable** | **Control_All_ (*n*= 85)** | **Intervention_All_ (*n*=** **74)** |
| --- | --- | --- |
| **QT-prolonging drugs prescribed (per patient in %)** | | |
| Amitriptyline | 5 (5.9%) | 2 (2.7%) |
| Aripiprazole | 4 (4.7%) | 4 (5.4%) |
| Chlorprothixene | 2 (2.4%) | 1 (1.4%) |
| Citalopram | 5 (5.9%) | 3 (4.1%) |
| Clozapine | 1 (1.2%) | 0 |
| Doxepine | 3 (3.5%) | 3 (4.1%) |
| Escitalopram | 10 (11.8%) | 6 (8.1%) |
| Fluoxetine | 2 (2.4%) | 1 (1.4%) |
| Lithium | 4 (4.7%) | 0 |
| Mirtazapine | 9 (10.6%) | 5 (6.8%) |
| Olanzapine | 3 (3.5%) | 3 (4.1%) |
| Pipamperone | 1 (1.2%) | 1 (1.4%) |
| Promethazine | 8 (9.4%) | 10 (13.5%) |
| Prothipendyl | 5 (5.9%) | 1 (1.4%) |
| Quetiapine | 13 (15.3%) | 9 (12.2%) |
| Risperidone | 1 (1.2%) | 0 |
| Sertraline | 7 (8.2%) | 14 (18.9%) |
| Trimipramine | 3 (3.5%) | 4 (5.4%) |
| Venlafaxine | 13 (15.3%) | 4 (5.4%) |
| **Number of psychotropic drugs prescribed with risk for QTc prolongation based on AZCERT-categories (per patient in %)** | | |
| Drug prescriptions with known risk | 17 (20%) | 10 (13.5%) |
| Drug prescriptions possible risk | 50 (58.8%) | 39 (52.7%) |
| Drug prescriptions with conditional risk | 34 (40%) | 34 (45.9%) |

**Supplementary Table 7.** QTc prolonging somatic drugs and their AZCERT-categories prescribed to patients in the control (CG; *n*= 85) and intervention groups (IG; *n* = 74) at admission.

| **Variable** | **Control_All_ (*n*= 85)** | **Intervention_All_ (*n*=** **74)** |
| --- | --- | --- |
| **QT-prolonging drugs prescribed (per patient in %)** | | |
| Chinine sulfate | 0 | 1 (1.4%) |
| Esomeprazole | 0 | 2 (2.7%) |
| Furosemide | 1 (1.2%) | 0 |
| Hydrochlorothiazide | 1 (1.2%) | 2 (2.7%) |
| Indapamide | 2 (2.4%) | 0 |
| Ivabradine | 0 | 1 (1.4%) |
| Loperamide | 1 (1.2%) | 0 |
| Metoclopramide | 1 (1.2%) | 1 (1.4%) |
| Omeprazole | 0 | 2 (2.7%) |
| Ondansetrone | 1 (1.2%) | 0 |
| Pantoprazole | 13 (15.3%) | 7 (9.5%) |
| Tizanidine | 0 | 1 (1.4%) |
| Torasemide | 1 (1.2%) | 2 (2.7%) |
| Tramadole | 0 | 2 (2.7%) |
| **Number of psychotropic drugs prescribed with risk for QTc prolongation based on AZCERT-categories (per patient in %)** | | |
| Drug prescriptions with known risk | 1 (1.2%) | 0 |
| Drug prescriptions with possible risk | 0 | 3 (4.1%) |
| Drug prescriptions with conditional risk | 20 (23.5%) | 18 (24.3%) |

**Supplementary Table 8.** Results from the satisfaction survey in July 2024 among physicians and nurses.

| **Employees working on the study wards between**  **01-06/2024** | **Psychiatric physicians** | **Nurses** | **Overall** |
| --- | --- | --- | --- |
| Total (% of all employees) | 15 (25%) | 45 (75%) | 60 (100%) |
| Survey participants (% of all respondents) | 4 (30.8%) | 9 (69.2%) | 13 (21.7%) |
| Return rate (%) | 26.7% | 20% | 21.7% |
| **Responses** | | | |
| *Satisfaction with interdisciplinary QTc monitoring* | | | |
| How useful were the pharmacist’s recommendations for the treatment of your patients? [No. of responses (%), median (IQR)]  *Very useful: 1; Useful: 2; Partly useful: 3; Mostly not useful: 4; Not useful: 5* | 4 (100%)  Very useful (0.25) | 7 (77.8%)  Useful (0.5) | 11 (84.6%)  Useful (1) |
| Satisfaction with interdisciplinary treatment of patients prescribed QT prolonging drugs [No. of responses (%), median (IQR)]* | 4 (100%)  Very satisfied (0.25) | 6 (66.7%)  Very satisfied (0) | 10 (76.9%)  Very satisfied (0) |
| Satisfaction with pharmaceutical contribution to interdisciplinary treatment of patients prescribed QT prolonging drugs [No. of responses (%), median (IQR)]* | 4 (100%)  Very satisfied (0) | 5 (38.5%)  Very satisfied (0) | 9 (69.2%)  Very satisfied (0) |
| Desire for continuation of pharmaceutical contribution as during study period [“Yes”: *n* (% of respondents)] | 3 (75%) | 7 (77.8%) | 10 (76.9%) |
| *Resources for ECG monitoring* | | | |
| Sufficiency of personnel resources [No. of responses (%), median (IQR)]^†^ | 4 (100%)  Rather insufficient (0.25) | 8 (88.9%)  Rather insufficient (2) | 12 (92.3%)  Rather insufficient (1.25) |
| Sufficient number of nurses trained in ECG writing [No. of responses (%), median (IQR)]^†^ | 4 (100%)  Between rather sufficient and rather insufficient: 2.5 (1) | 6 (66.7%)  Rather sufficient (1.5) | 10 (76.9%)  Rather sufficient (1) |
| Sufficient number of existing ECG devices [No. of responses (%), median (IQR)]^†^ | 3 (75%)  Rather insufficient (1.5) | 8 (88.9%)  Between sufficient and rather sufficient: 1.5 (1.25) | 11 (84.6%)  Rather sufficient (2) |

No.: Number. **Very satisfied: 1, Satisfied: 2, Rather not satisfied: 3, Not satisfied: 4.* ^†^*Sufficient: 1, Rather sufficient: 2, Rather insufficient: 3; Insufficient: 4.*

**Supplementary Table 9.** Free text questions asked in the employee satisfaction survey in July 2024 among physicians and nurses and corresponding responses.

| **Free text question asked in the survey** | **Responses received in July 2024** |
| --- | --- |
| What other recommendations have you received from pharmacists in the last 6 months? | No responses received |
| What other pharmaceutical or interdisciplinary support measures would you like to see? | - Irregular participation of pharmacists in senior physician rounds, e.g., once per quarter for interprofessional exchange* |
| What other feedback or suggestions for improvement do you have regarding interdisciplinary collaboration in QTc monitoring during psychopharmacotherapy? | - Feedback on calculation of QTc time (which formula was used for QT-correction) |
| What other feedback or suggestions for improvement do you have for the pharmacy in general? | - I think it would be useful to have regular discussions about medication, its effects, and its usefulness in relation to patients and their diagnoses.* |

**same participant*

# Reporting checklist for randomised trial

Based on the CONSORT guidelines.

Schulz KF, Altman DG, Moher D, for the CONSORT Group. CONSORT 2010 Statement: updated guidelines for reporting parallel group randomised trials

|  |  | Reporting Item | Page Number |
| --- | --- | --- | --- |
| **Title and Abstract** |  |  |  |
| Title | [#1a](https://www.goodreports.org/reporting-checklists/consort/info/#1a) | Identification as a randomized trial in the title. | Title |
| Abstract | [#1b](https://www.goodreports.org/reporting-checklists/consort/info/#1b) | Structured summary of trial design, methods, results, and conclusions | Abstract |
| **Introduction** |  |  |  |
| Background and objectives | [#2a](https://www.goodreports.org/reporting-checklists/consort/info/#2a) | Scientific background and explanation of rationale | 1. Introduction |
| Background and objectives | [#2b](https://www.goodreports.org/reporting-checklists/consort/info/#2b) | Specific objectives or hypothesis | 1. Introduction |
| **Methods** |  |  |  |
| Trial design | [#3a](https://www.goodreports.org/reporting-checklists/consort/info/#3a) | Description of trial design (such as parallel, factorial) including allocation ratio. | 2.1 Trial design |
| Trial design | [#3b](https://www.goodreports.org/reporting-checklists/consort/info/#3b) | Important changes to methods after trial commencement (such as eligibility criteria), with reasons | n/a |
| Participants | [#4a](https://www.goodreports.org/reporting-checklists/consort/info/#4a) | Eligibility criteria for participants | 2.2.1 Inclusion and exclusion criteria |
| Participants | [#4b](https://www.goodreports.org/reporting-checklists/consort/info/#4b) | Settings and locations where the data were collected | 2.2.2 Setting |
| Interventions | [#5](https://www.goodreports.org/reporting-checklists/consort/info/#5) | The experimental and control interventions for each group with sufficient details to allow replication, including how and when they were actually administered | 2.3 Interventions |
| Outcomes | [#6a](https://www.goodreports.org/reporting-checklists/consort/info/#6a) | Completely defined prespecified primary and secondary outcome measures, including how and when they were assessed | 2.4 Outcomes |
| Outcomes | [#6b](https://www.goodreports.org/reporting-checklists/consort/info/#6b) | Any changes to trial outcomes after the trial commenced, with reasons | n/a |
| Sample size | [#7a](https://www.goodreports.org/reporting-checklists/consort/info/#7a) | How sample size was determined. | 2.7 Sample size |
| Sample size | [#7b](https://www.goodreports.org/reporting-checklists/consort/info/#7b) | When applicable, explanation of any interim analyses and stopping guidelines | n/a |
| Randomization – Sequence generation | [#8a](https://www.goodreports.org/reporting-checklists/consort/info/#8a) | Method used to generate the random allocation sequence. | 2.8 Randomization |
| Randomization - Sequence generation | [#8b](https://www.goodreports.org/reporting-checklists/consort/info/#8b) | Type of randomization; details of any restriction (such as blocking and block size) | 2.8 Randomization |
| Randomization - Allocation concealment mechanism | [#9](https://www.goodreports.org/reporting-checklists/consort/info/#9) | Mechanism used to implement the random allocation sequence (such as sequentially numbered containers), describing any steps taken to conceal the sequence until interventions were assigned | 2.8 Randomization |
| Randomization - Implementation | [#10](https://www.goodreports.org/reporting-checklists/consort/info/#10) | Who generated the allocation sequence, who enrolled participants, and who assigned participants to interventions | 2.8 Randomization |
| Blinding | [#11a](https://www.goodreports.org/reporting-checklists/consort/info/#11a) | If done, who was blinded after assignment to interventions (for example, participants, care providers, those assessing outcomes) and how. | 2.8 Randomization |
| Blinding | [#11b](https://www.goodreports.org/reporting-checklists/consort/info/#11b) | If relevant, description of the similarity of interventions | n/a |
| Statistical methods | [#12a](https://www.goodreports.org/reporting-checklists/consort/info/#12a) | Statistical methods used to compare groups for primary and secondary outcomes | 2.9 Statistical methods |
| Statistical methods | [#12b](https://www.goodreports.org/reporting-checklists/consort/info/#12b) | Methods for additional analyses, such as subgroup analyses and adjusted analyses | 2.9 Statistical methods |
| **Results** |  |  |  |
| Participant flow diagram (strongly recommended) | [#13a](https://www.goodreports.org/reporting-checklists/consort/info/#13a) | For each group, the numbers of participants who were randomly assigned, received intended treatment, and were analysed for the primary outcome | 3.1 Participant flow and recruitment, Fig. 2 |
| Participant flow | [#13b](https://www.goodreports.org/reporting-checklists/consort/info/#13b) | For each group, losses and exclusions after randomization, together with reason | 3.1 Participant flow and recruitment, Fig. 2 |
| Recruitment | [#14a](https://www.goodreports.org/reporting-checklists/consort/info/#14a) | Dates defining the periods of recruitment and follow-up | 3.1 Participant flow and recruitment |
| Recruitment | [#14b](https://www.goodreports.org/reporting-checklists/consort/info/#14b) | Why the trial ended or was stopped | 3.1 Participant flow and recruitment |
| Baseline data | [#15](https://www.goodreports.org/reporting-checklists/consort/info/#15) | A table showing baseline demographic and clinical characteristics for each group | 3.1 Participant flow and recruitment, Tab. 1, Suppl. Tab. 4-5 |
| Numbers analysed | [#16](https://www.goodreports.org/reporting-checklists/consort/info/#16) | For each group, number of participants (denominator) included in each analysis and whether the analysis was by original assigned groups | 3.1 Participant flow and recruitment |
| Outcomes and estimation | [#17a](https://www.goodreports.org/reporting-checklists/consort/info/#17a) | For each primary and secondary outcome, results for each group, and the estimated effect size and its precision (such as 95% confidence interval) | 3.2 Primary outcome: Mean change in QTc interval, 3.3 Secondary outcomes |
| Outcomes and estimation | [#17b](https://www.goodreports.org/reporting-checklists/consort/info/#17b) | For binary outcomes, presentation of both absolute and relative effect sizes is recommended | 3.2 Primary outcome: Mean change in QTc interval, 3.3 Secondary outcomes |
| Ancillary analyses | [#18](https://www.goodreports.org/reporting-checklists/consort/info/#18) | Results of any other analyses performed, including subgroup analyses and adjusted analyses, distinguishing pre-specified from exploratory | 3.2 Primary outcome: Mean change in QTc interval, 3.3 Secondary outcomes |
| Harms | [#19](https://www.goodreports.org/reporting-checklists/consort/info/#19) | All important harms or unintended effects in each group (For specific guidance see CONSORT for harms) | 3.3.1 Secondary outcomes |
| **Discussion** |  |  |  |
| Limitations | [#20](https://www.goodreports.org/reporting-checklists/consort/info/#20) | Trial limitations, addressing sources of potential bias, imprecision, and, if relevant, multiplicity of analyses | 4.1 Strengths and limitations |
| Generalisability | [#21](https://www.goodreports.org/reporting-checklists/consort/info/#21) | Generalisability (external validity, applicability) of the trial findings | 4. Discussion |
| Interpretation | [#22](https://www.goodreports.org/reporting-checklists/consort/info/#22) | Interpretation consistent with results, balancing benefits and harms, and considering other relevant evidence | 4. Discussion |
| Registration | [#23](https://www.goodreports.org/reporting-checklists/consort/info/#23) | Registration number and name of trial registry | Abstract, 2.1 Trial design |
| **Other information** |  |  |  |
| Interpretation | [#22](https://www.goodreports.org/reporting-checklists/consort/info/#22) | Interpretation consistent with results, balancing benefits and harms, and considering other relevant evidence | 4. Discussion |
| Registration | [#23](https://www.goodreports.org/reporting-checklists/consort/info/#23) | Registration number and name of trial registry | Abstract, 2.1 Trial design |
| Protocol | [#24](https://www.goodreports.org/reporting-checklists/consort/info/#24) | Where the full trial protocol can be accessed, if available | n/a |
| Funding | [#25](https://www.goodreports.org/reporting-checklists/consort/info/#25) | Sources of funding and other support (such as supply of drugs), role of funders | 7. Funding, None |

The CONSORT checklist is distributed under the terms of the Creative Commons Attribution License CC-BY. This checklist can be completed online using <https://www.goodreports.org/>, a tool made by the [EQUATOR Network](https://www.equator-network.org) in collaboration with [Penelope.ai](https://www.penelope.ai)
